# Effect of sedation with dexmedetomidine or propofol on gastrointestinal motility in lipopolysaccharide-induced endotoxemic mice

**DOI:** 10.1186/s12871-020-01146-z

**Published:** 2020-09-07

**Authors:** Haiqing Chang, Shuang Li, Yansong Li, Hao Hu, Bo Cheng, Jiwen Miao, Hui Gao, Hongli Ma, Yanfeng Gao, Qiang Wang

**Affiliations:** 1 Department of Anesthesiology & Center for Brain Science, The First Affiliated Hospital of Xi’an Jiaotong University, Xi’an, 710061 Shaanxi China; 2 Department of Pharmacology, School of Basic Medical Sciences, Health Science Center, Xi’an Jiaotong University, Xi’an, 710061 Shaanxi China

**Keywords:** Endotoxemia, Dexmedetomidine, Gastrointestinal motility, ICU, Propofol, Sedation, Sepsis

## Abstract

**Background:**

Sepsis often accompanies gastrointestinal motility disorder that contributes to the development of sepsis in turn. Propofol and dexmedetomidine, as widely used sedatives in patients with sepsis, are likely to depress gastrointestinal peristalsis. We queried whether propofol or dexmedetomidine, at sedative doses, aggravated sepsis-induced ileus.

**Methods:**

Sedative/Anesthetic Scores and vital signs of lipopolysaccharide (LPS)-induced endotoxemic mice were measured during sedation with propofol or dexmedetomidine. Endotoxemic mice were divided into 10% fat emulsion, propofol, saline, and dexmedetomidine group. The gastric emptying, small intestinal transit, tests of colonic motility, gastrointestinal transit and whole gut transit were evaluated at 15 mins and 24 h after intraperitoneal injection of sedatives/vehicles respectively.

**Results:**

40 mg·kg^− 1^propofol and 80 μg·kg^− 1^ dexmedetomidine induced a similar depth of sedation with comparable vital signs except that dexmedetomidine strikingly decreased heart rate in endotoxemic mice. Dexmedetomidine markedly inhibited gastric emptying (*P* = 0.006), small intestinal transit (*P* = 0.006), colonic transit (*P* = 0.0006), gastrointestinal transit (*P* = 0.0001) and the whole gut transit (*P* = 0.034) compared with the vehicle, whereas propofol showed no depression on all parts of gastrointestinal motility 15 mins after administration. The inhibitive effects of dexmedetomidine in these tests vanished 24 h after the administration.

**Conclusions:**

Deep sedation with dexmedetomidine, but not propofol, significantly inhibited gastrointestinal peristalsis in endotoxemic mice while the inhibitory effect disappeared 24 h after sedation. These data suggested that both propofol and dexmedetomidine could be applied in septic patients while dexmedetomidine should be used cautiously in patients with cardiac disease or ileus.

## Background

Sepsis has become a life-threatening organ dysfunction with up to 26% mortality in the last decade. It is estimated that there are 19.4 million cases of severe sepsis worldwide, with potentially 5.3 million deaths annually [1]. Impairment of intestinal motility is an inevitable complication of sepsis and sepsis has been identified as one of the risk factors for developing the gastrointestinal (GI) motility problems [2]. In turn, inhibition of propulsive intestinal motility predisposes to gut-derived microbial translocation, which plays a pivotal role in the development of sepsis [3]. It is suggested that a vicious circle might be created by sepsis and GI motility. Thus, in sepsis, GI motility disorder demands our ongoing attention and research.

Severe septic patients who need mechanical ventilation account for 10–20% of all admissions to the intensive care unit (ICU) [4, 5]. Propofol and dexmedetomidine are recommended sedatives for septic patients [6]. Impairment of intestinal peristalsis by sedatives is a major side effect, however, scant attention has been given to it so far [7]. Previous studies demonstrated that symptoms of impaired GI transit such as constipation and feed intolerance occurred in up to 50% of mechanically ventilated patients in ICU and these patients had a longer ICU stay [8, 9]. Thus, we need to devote more attention to the effect of sedatives on GI motility in sepsis.

Propofol as an intravenous anesthetic agent gained US FDA approval for sedation in ICU in 1993 [10]. Some studies showed propofol inhibited gut peristalsis and others showed no alteration on the amplitude of CMMCs in the distal colon and GI transit with propofol [11, 12]. However, extensive researches suggested dexmedetomidine as a popular sedative inhibited GI peristalsis [13, 14]. It is preferable to use a sedative that has fewer inhibitory effects on GI transit, but there is a paucity of data describing this topic in sepsis and there are limited methods that could comprehensively evaluate the motility of all parts of the GI tract in humans. Thus, we sought to examine whether propofol and dexmedetomidine, at sedative doses, can inhibit on GI motility in endotoxemic mice and to compare their differences?

## Methods

### Animals

Eight–ten weeks old C57BL/6 J male mice were supplied by the Laboratory Animal Center of Xi’an Jiaotong University. A standard laboratory diet was given to the mice in a controlled environment (light: dark: 1:1, the cycle starts at 8 Am every day. All animal protocols followed Animal Research: Reporting of In Vivo Experiments (ARRIVE) Guidelines and were approved by the Institutional Animal Care and Use Committee of Xi’an Jiaotong University. There were no adverse events related to the animals throughout the experiment.

### Drugs

Lipopolysaccharide (LPS), Evans blue, methylcellulose, 70 kDa fluorescein isothiocyanate conjugated dextran were bought from Sigma-Aldrich (St Louis, MO, USA). Propofol (Diprivan®, AstraZeneca, London, British), dexmedetomidine (Yangtze River Pharmaceutical Group, Taizhou, Jiangsu, China), isoflurane (RWD Life Science, Shenzhen, Guangdong, China) and 10% fat emulsion (Intralipid®, Fresenius Kabi, Wuxi, Jiangsu, China) were used in present study.

### Experimental protocol

First of all, 5 mg·kg^− 1^ LPS was applied to build the endotoxemic model. For confirming doses of propofol and dexmedetomidine that could induce similar depth of sedative level, sedative/anesthetic scores of endotoxemic mice was evaluated after those mice were injected i.p. using different doses of propofol and dexmedetomidine.

Then, the pulse oxygen saturation, respiratory rate, heart rate and systolic blood pressure were compared between the mice receiving 40 mg.kg^− 1^ propofol and those receiving 80 μg·kg^− 1^ dexmedetomidine.

**Fig. 1:**
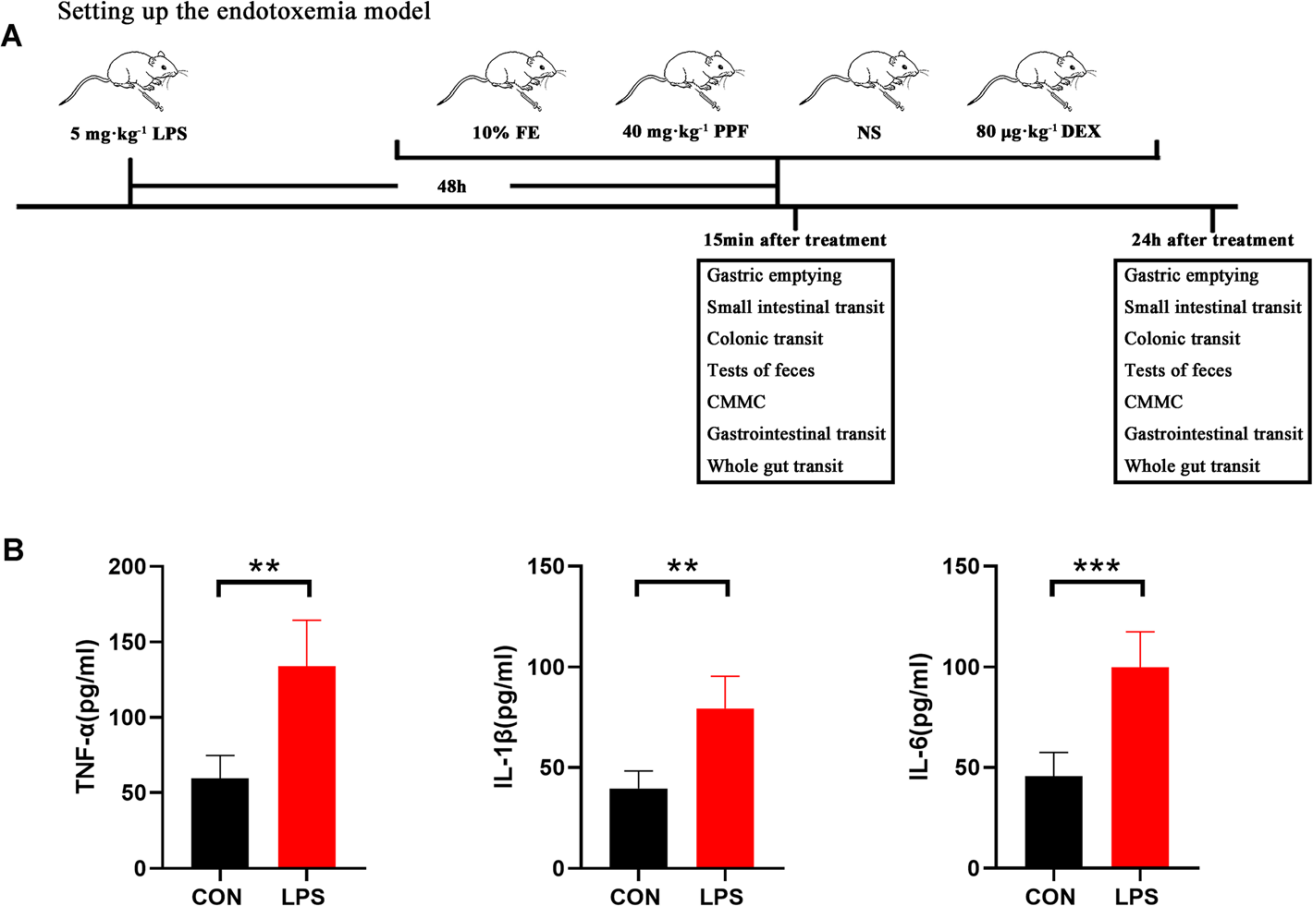
Protocol of assessing gastrointestinal motility and detection of IL-6, TNF-α, and IL-1β serum levels. *n* = 5 per group. **a**. Firstly, lipopolysaccharide (LPS, 5 mg·kg^− 1^) was used to set up the endotoxemia model. Then the endotoxemic mice were randomized to four groups 48 h after model establishment, and the following drugs were injected intraperitoneally: 10% fat emulsion, 40 mg·kg^− 1^ propofol, normal saline, 80 μg·kg^− 1^ dexmedetomidine. Next, gastric emptying, small intestinal transit, colonic transit, tests of feces, colonic migrating motor complexes, gastrointestinal transit, and whole gut transit were performed 15 mins after injection of sedatives/vehicles. Finally, the same tests were conducted 24 h after the injection of sedatives/vehicles. **b**. The mice in the endotoxemia group have much higher IL-6, TNF-α, and IL-1β serum levels than those mice in the control group. Data were expressed as mean ± SD and analysed by unpaired t test. **P < 0.01, **P < 0.001. CON, control. LPS, lipopolysaccharide. FE, fat emulsion. PPF, propofol. NS, normal saline. DEX, dexmedetomidine

Finally, as described in Fig. 1a, motility tests of different gastrointestinal section were conducted 15 mins and 24 h after the injection of sedatives/vehicles again respectively.

### Endotoxemia model

To set up the endotoxemia model, mice would receive a single intraperitoneal injection of 5 mg·kg^− 1^ LPS in 0.5 mL 48 h before injection of the sedatives/solvents [15].

### Measurement of serum IL-6, TNF-α, and IL-1β levels

A total of 500 μl of blood was collected 48 h after intraperitoneal administration of 5 mg·kg-1 LPS or equal volume saline. Following incubation for 1 h, the blood was centrifuged at 2000 g for 10 mins to obtain the serum. Serum IL-6, TNF-α, and IL-1β levels were measured using enzyme-linked immunosorbent assay (ELISA) kits kit from Assay Designs. ELISA kits of IL-6, TNF-α, and IL-1β were purchased from Beyotime Biotechnology (Shanghai, China).

### Euthanasia

In some of the following tests of gastrointestinal motility, the mice were sacrificed to examine the gastrointestinal motor function. According to the 2013 AVMA (American Veterinary Medical Association) Guidelines for the Euthanasia of Animals, animals were euthanized via a continuous 5% isoflurane exposure until 1 min after the breath stop. Then gastrointestinal tissue was obtained in these tests.

### Sedative/anesthetic scores

Mice were scored every 5 mins after sedatives/vehicles application: wakefulness (score 0): spontaneous locomotor activity in 1 min observation; light sedation (0.2): no spontaneous locomotion in 1 min observation; deep sedation (0.4): no motor response when placed on a grid inclined (45°) with the head down during 30s observation; light anesthesia (0.6): no righting reflex during 30s observation; moderate anesthesia (0.8): no paw withdrawal reflex and deep anesthesia (1.0): no eye blink reflex [16].

### Monitoring of vital signs

Vital signs were measured in 5-min intervals during a 35 mins period. Heart rate and breaths were measured by the BL-420F Data Acquisition & Analysis System (Techman Software Co. LTD, Chengdu, China). Systolic pressure was measured by the tail-cuff system (BP-2000 Blood Pressure Analysis System, Visitech Systems, Apex, NC). Oxygen saturation was measured by Radical 7 (Masimo Corporation, Irvine, USA). Vital signs were recorded at 5 mins after sedation since stable parameters can be gained only when mice were relatively calm and stationary.

### Measurement of gastric emptying (GE) and small intestinal transit (SIT)

Overnight fasting mice were given intragastrically 0.1 ml solution containing 5% Evans blue and 1.5% methylcellulose and were sacrificed 15 mins later. The migrating distance of Evans blue and the total length of the small intestine were measured and transit was expressed in %. The stomach was minced and diluted. And the absorbance of each sample was read at a wavelength of 565 nm (A565). The stomach obtained from a mouse sacrificed immediately after orogastric administration of Evans blue served as a standard (reference stomach). The percentage of GE was calculated by the formula %GE = [(A565reference - A565sample)/A565 reference] × 100% [17].

### Colonic transit

Briefly, mice were fasted overnight. A 2 mm glass bead was inserted 2 cm deep into the distal colon after mice were anesthetized using 2% isoflurane. The bead expulsion latency was measured after the recovery of righting reflex [18].

### Tests of feces

Mice were housed individually without food for an hour. Fecal pellet output was collected during this period, and numbers and wet weight of feces were recorded. Pellets were dried at 60 °C in the oven overnight, then the dried pellets were weighed right after [19].

### Video imaging of colonic migrating motor complexes (CMMCs)

Mice were sacrificed 15 mins after injection of sedatives/solvents. The entire colon was removed and put into Kerbs solution (NaCl 120, KCl 4.7, CaCl_2_ 2.4, MgSO_4_ 1.2, NaHCO_3_ 24.5, KH_2_PO_4_ 1.0 and glucose 5.6 in mM, pH 7.4). Then, the colon was mounted to allow spontaneous motor patterns to be imaged for the construction of spatiotemporal maps. The contractile activity was recorded with a Logitech Pro camera and video data were processed with MATLAB® (R2018a, version 9.4). Spatiotemporal maps of the diameter at each point along the proximo-distal length of the colon were constructed and used to quantify the frequency of CMMCs as well as the velocity and length of propagation of CMMCs [20].

### Gastrointestinal transit (GIT)

GI transit was examined by calculating the Geometric Center (GC) from the average distribution of a non-absorbable fluorescent marker along the GI tract. FITC-dextran was dissolved at a concentration of 5 mg·ml^−1^with 0.5% methylcellulose and was given into the stomach (0.1 ml). GI tract of sacrificed mice was harvested 45 mins later and cut averagely into stomach, small bowel (10 segments of equal length), cecum and colon (3 segments of equal length). Tissues were minced, vortexed and centrifuged with 1 ml saline. Supernatants were loaded into a 96 well plate, and the fluorescent signal was read (CytofluorTM plate reader; excitation 492 nm, emission 518 nm). Geometric Center (GC) was calculated as: ∑(S1 x 1 + S2 x 2 + …S15 x 15), where S was the fraction of the total signal detected in each of the 15 segments [21].

### Whole gut transit time (WGTT)

After overnight fasting, mice received intragastrically 0.1 ml of a solution containing Evans blue. Time was recorded from the administration of oral maker to the first appearance of a blue pellet [22].

### Statistical analysis

Statistical analyses were performed with Prism 8.0 (GraphPad, San Diego, CA, USA). All of the mice were randomly grouped and tagged, and the statistician was blind to the experimental performer. Shorter migration of maker, longer transit time, less defecation and smaller GC were considered as worse motility. Results are presented as mean ± SD (standard deviation). Data were evaluated for normal distribution and homogeneity of variance, then analysed by one-way ANOVA (between-group differences were detected with Tukey post hoc tests) or Kruskal–Wallis test (followed by Dunn’s multiple comparisons test with Bonferroni correction). Two-way repeated-measures ANOVA was used to analyse vital signs (followed by Sidak’s post hoc test for multiple comparisons where applicable). An unpaired t test was used to analyse serum IL-6, TNF-α, and IL-1β levels.. Statistical significance was assigned at *P* < 0.05.

## Results

### Propofol and dexmedetomidine induced dose-dependent sedation in endotoxemic mice

**Fig. 2:**
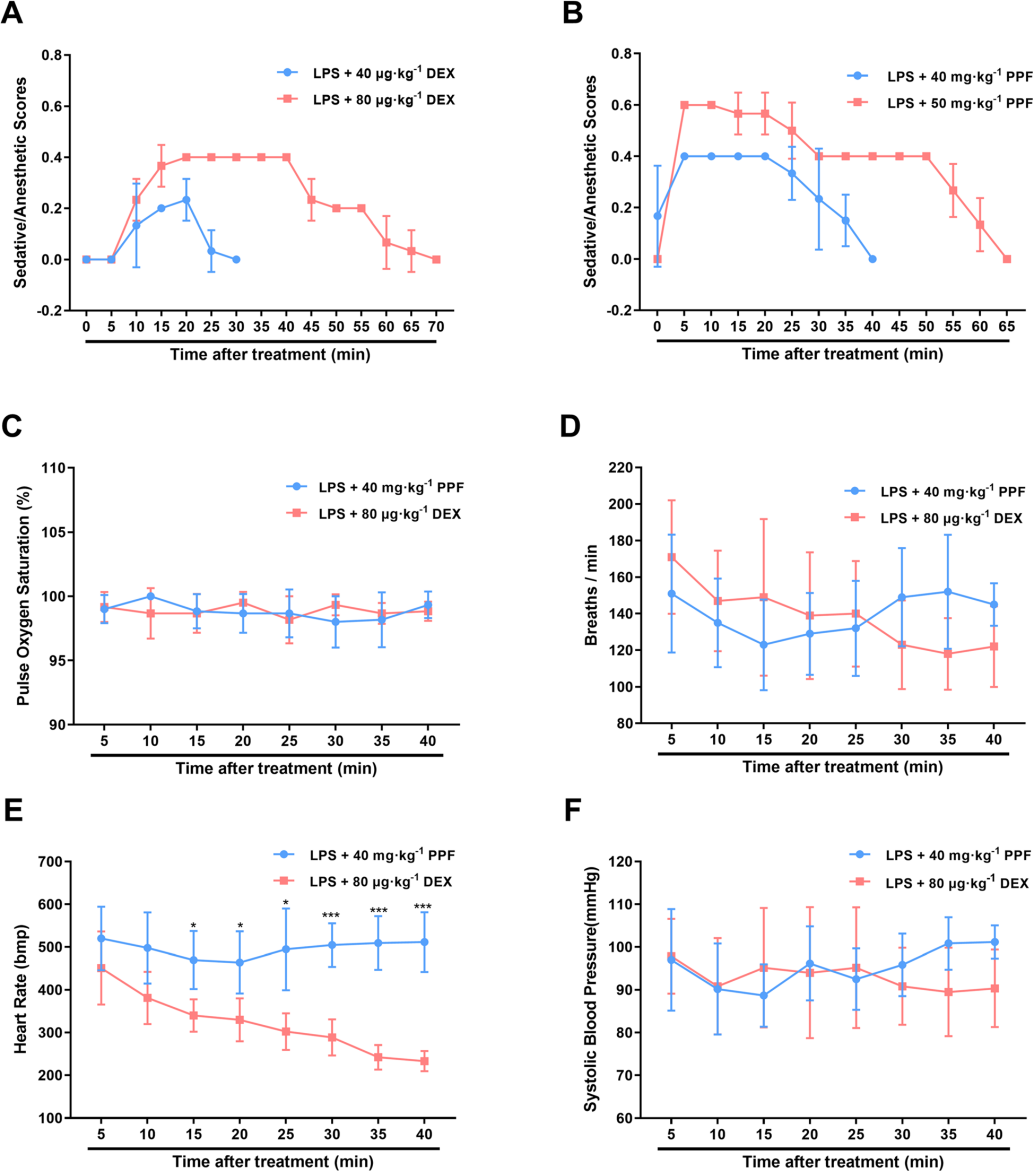
Depth of sedation and vital signs of endotoxemic mice after administration of propofol or dexmedetomidine. *n* = 6 per group. **a**. Does-dependent sedative depth of endotoxemic mice accepting 40 μg·kg^− 1^ and 80 μg·kg^− 1^ dexmedetomidine. **b**. Does-dependent sedative depth of endotoxemic mice accepting 40 mg.kg^− 1^ and 50 mg.kg^− 1^ propofol. **c-f**. The mice with administration of 40 mg·kg^− 1^ propofol and 80 μg·kg^− 1^ dexmedetomidine had similar pulse oxygen saturation percentage, respiratory rate, and systolic blood pressure over time while dexmedetomidine strikingly decreased heart rate. Vital signs were recorded from 5 mins to 40 mins after sedation. Data were expressed as mean ± SD and analysed by two-way repeated-measures ANOVA. ^*^P < 0.05, ^***^P < 0.001, LPS + 80 μg·kg^− 1^ DEX vs LPS + 40 mg·kg^−1^PPF. LPS, lipopolysaccharide. PPF, propofol. DEX, dexmedetomidine

Firstly, we measured the serum levels of IL-6, TNF-α, and IL-1β to confirm the successful establishment of the endotoxemia model (Fig. 1b). Then the depth of sedation of mice with different doses of propofol and dexmedetomidine was evaluated. We found both propofol and dexmedetomidine could induce dose-dependent sedative levels. 40 mg·kg^− 1^ propofol and 80 μg·kg^− 1^ dexmedetomidine produced a comparable deep sedative level 15 mins after injection and we used these doses to perform the following tests (Fig. 2a, b).

### Vital signs of endotoxemic mice during sedation with propofol and dexmedetomidine

Vital signs of sedative endotoxemic mice were measured for 35 mins. It revealed that there was no statistical difference between endotoxemic mice with administration of 40 mg·kg^− 1^ propofol and 80 μg·kg^− 1^ dexmedetomidine for breaths *(P* = 0.920), oxygen saturation (*P* = 0.925), and systolic pressure (*P* = 0.608), while heart rate decreased strikingly from 10 mins after the dexmedetomidine treatment (*P* < 0.0001) (Fig. 2c-f).

### Dexmedetomidine, but not propofol delayed GE and SIT in endotoxemic mice

**Fig. 3:**
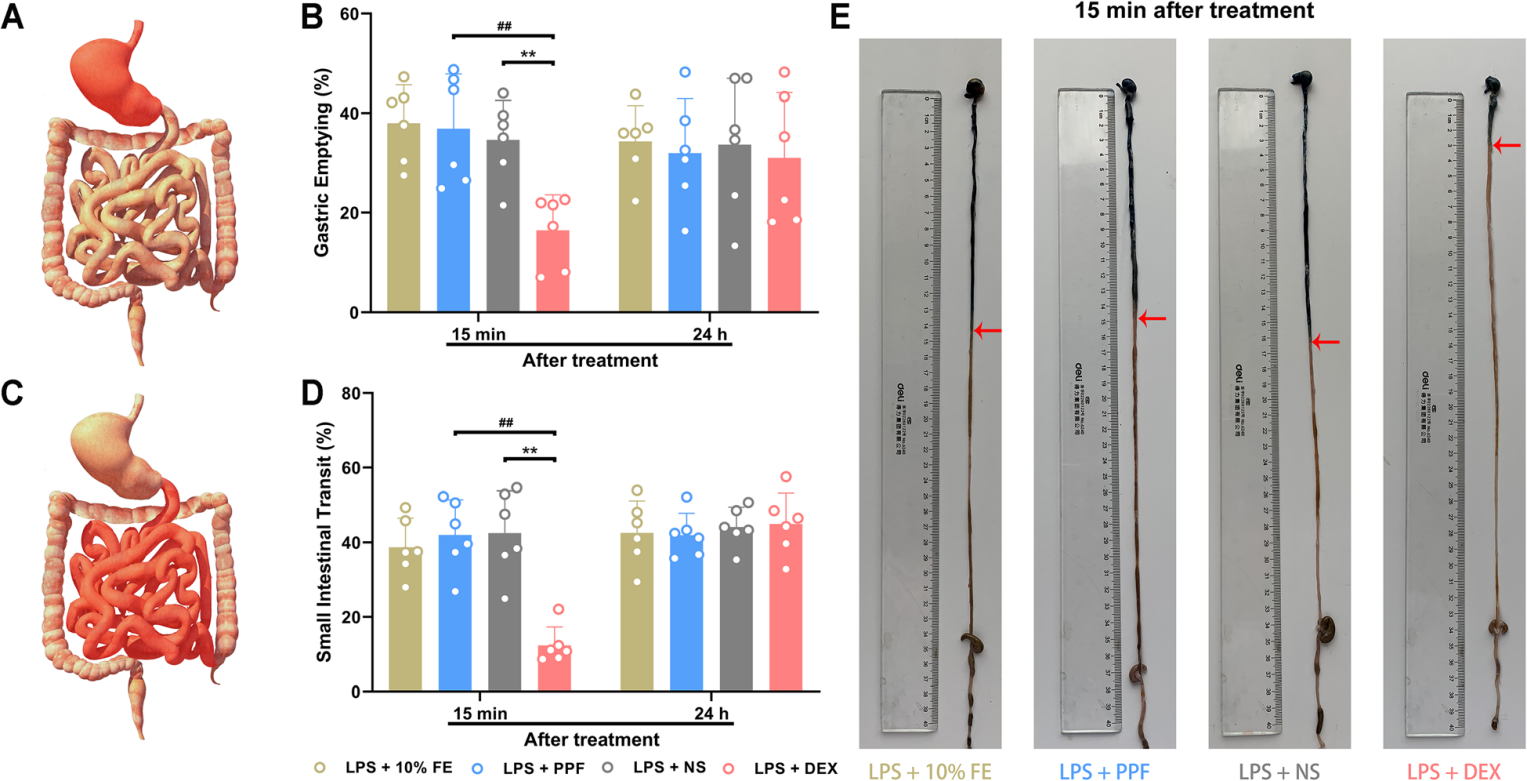
Effect of dexmedetomidine and propofol on upper gastrointestinal motility in endotoxemic mice. *n* = 6 per group. **a**. A schematic diagram of the stomach that examined in B. **b**. Dexmedetomidine inhibited gastric emptying of endotoxemic mice 15 mins after application compared with saline and propofol, and this depression disappeared 24 h after injection. **c**. A schematic diagram of the small intestine that examined in D. **d**. Dexmedetomidine but not propofol decreased small intestinal transit 15 mins after administration and this inhibition reversed 24 h after injection. **e**. Representative photographs showing small intestinal transit was measured by recording the migration of Evans blue (red arrows) 15 mins after application. Data were expressed as mean ± SD and analysed by one-way ANOVA or Kruskal–Wallis tests. ^**^P < 0.01, LPS + DEX vs LPS + NS; ^##^*P* < 0.01, LPS + DEX vs LPS + PPF. LPS, lipopolysaccharide. FE, fat emulsion. PPF, propofol. NS, normal saline. DEX, dexmedetomidine

Motility of the stomach and small intestine was examined (Fig. 3a, c). We found that GE and SIT were similar in 10% fat emulsion-treated and saline-treated mice. Dexmedetomidine inhibited GE of endotoxemic mice 15 mins after application compared with saline (16.4 ± 7.2% vs 34.7 ± 7.9%, *P* = 0.006) and propofol (16.4 ± 7.2% vs 36.9 ± 11.0%, *P* = 0.002) (Fig. 3b). Similarly, SIT was decreased by dexmedetomidine 15 mins after injection (see images in Fig. 3e), and statistically significant reduction was found as against mice with saline (12.3 ± 5.0% vs 42.5 ± 11.3%, *P* = 0.006) and with propofol (12.3 ± 5.0% vs 42.0 ± 9.4%, *P* = 0.008) (Fig. 3d). However, comparison of GE and SIT among mice with 10% fat emulsion to propofol revealed no significant difference. And dexmedetomidine had no inhibitory effect in GE and SIT 24 h after the application.

### Not propofol, but dexmedetomidine inhibited colonic transit and defecation in endotoxemic mice

**Fig. 4:**
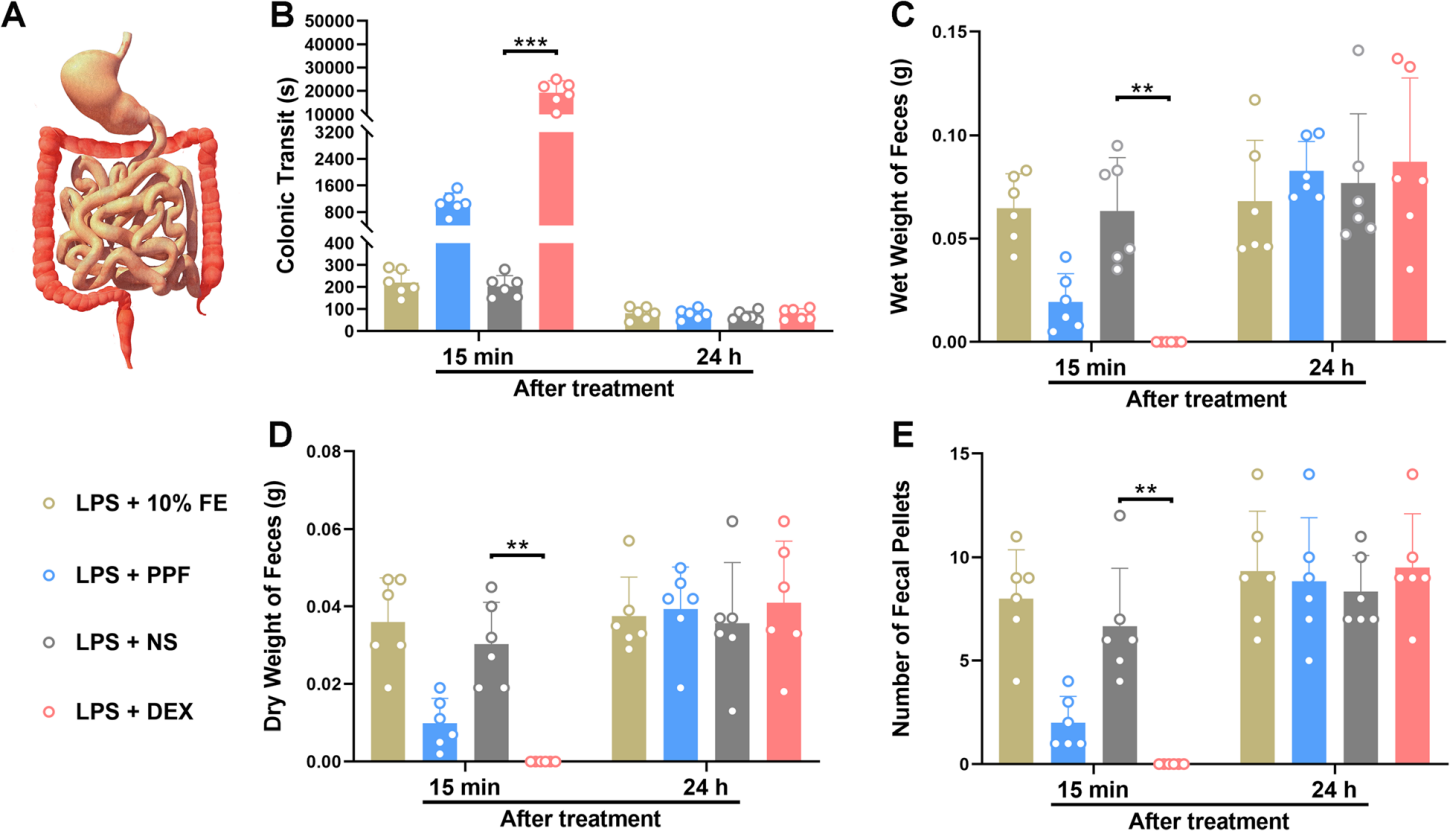
Effect of dexmedetomidine and propofol on colonic motility in vivo in endotoxemic mice. *n* = 6 per group. **a**. A schematic diagram of the colon. **b**. Dexmedetomidine had inhibitory effect on colonic transit time 15 mins after implement, and 24 h later, this inhibition didn’t exist anymore. **c-e**. Dexmedetomidine prevented excretion of feces. There was statistic difference between dexmedetomidine and saline in weight of feces, dry weight of feces and numbers of fecal pellets 15 mins after injection and there was no difference occurred 24 h after application between groups. Data were expressed as mean ± SD and analysed by one-way ANOVA or Kruskal–Wallis test. ^**^P < 0.01, ^***^P < 0.001, LPS + DEX vs LPS + NS. LPS, lipopolysaccharide. FE, fat emulsion. PPF, propofol. NS, normal saline. DEX, dexmedetomidine

Colonic motility was traced (Fig. 4a). There was no statistical difference between propofol group and 10% fat emulsion group, saline group and 10% fat emulsion group in colonic transit and defecation. Nevertheless, more time was required to expel glass beads in endotoxemic mice with dexmedetomidine (19,130 ± 5157 s) than those with saline (202 ± 49.6 s), *P* = 0.0006) (Fig. 4b). Fewer feces was excreted in an hour in the dexmedetomidine group, and statistic difference exited between dexmedetomidine-treated and saline-treated mice in the wet weight of feces (*P* = 0.001), dry weight of feces (*P* = 0.003) as well as the number of feces (*P* = 0.003) (Fig. 4c-e). This inhibition of dexmedetomidine on colonic transit and defecation reversed 24 h after implement.

### Dexmedetomidine, but not propofol suppressed CMMCs in endotoxemic mice

**Fig. 5:**
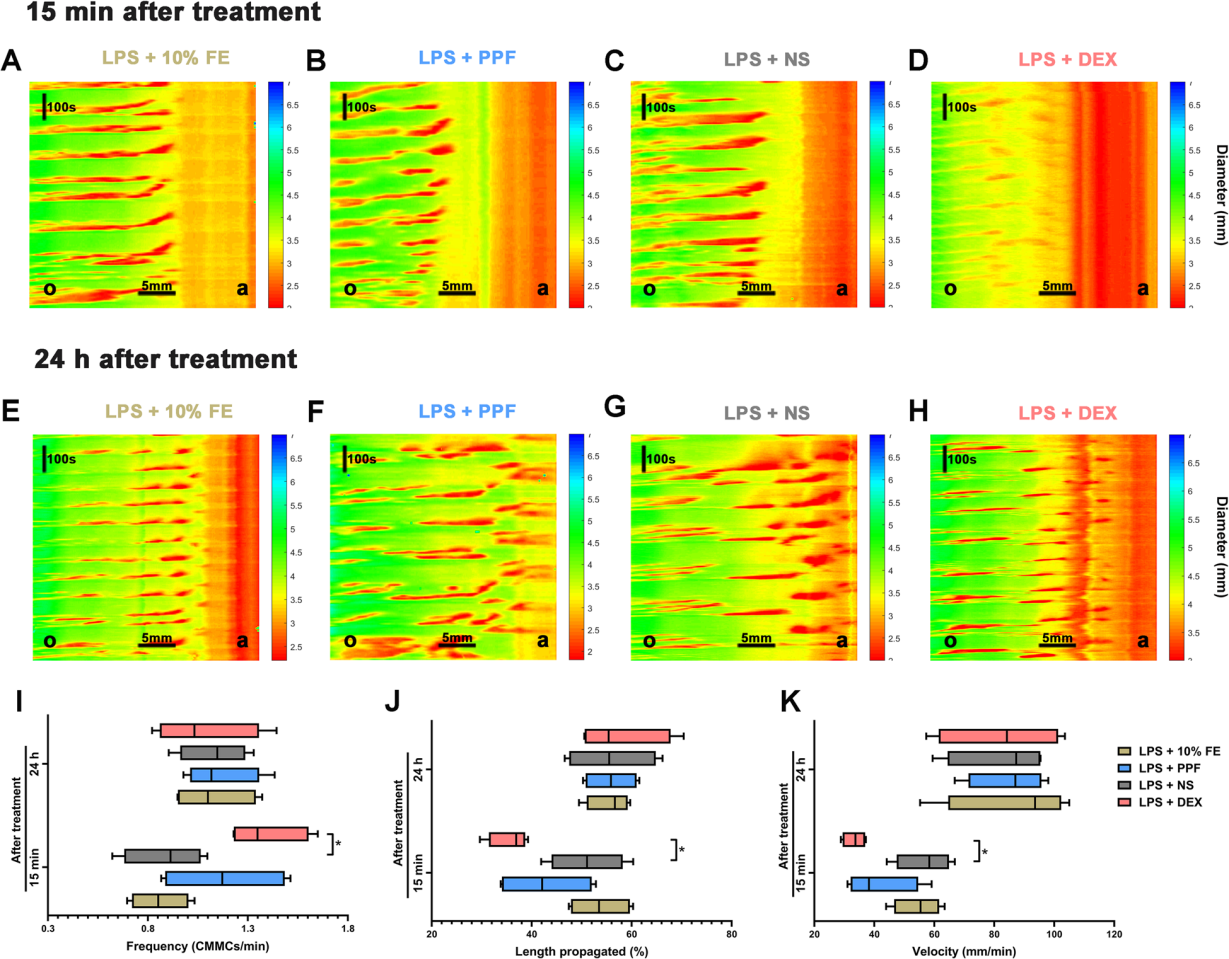
Effect of dexmedetomidine and propofol on CMMCs in endotoxemic mice. *n* = 4 per group. Typical spatiotemporal maps showed CMMCs in endotoxemic mice receiving 10% fat emulsion, propofol, saline, and dexmedetomidine 15 mins and 24 h after treatment in **a-d** and **e-h** respectively. The ordinate represents time, and the abscissa is indicative of spatial location from the oral end (O) to the anal end (A). The width of the gut (mm), representative of contractions, was pseudocolored. **i-k.** dexmedetomidine not propofol increased CMMCs frequency, shortened percentage of the length of propagation and slowed down velocity of propagation 15 mins after injection. Dexmedetomidine had no effect on CMMCs 24 h after injection. Data were expressed as median and interquartile ranges and analysed by one-way ANOVA. ^*^P < 0.05, LPS + DEX vs LPS + NS. LPS, lipopolysaccharide. FE, fat emulsion. PPF, propofol. NS, normal saline. DEX, dexmedetomidine. CMMCs, colonic migrating motor complexes

Spontaneous motility of isolated colon was recorded to check the suppressive effect of dexmedetomidine on colon could still be established in vitro. Spatiotemporal maps of contractile activity patterns were constructed 15 mins (Fig. 5a-d) and 24 h (Fig. 5e-h) after the treatment of sedatives/vehicles. CMMCs frequency was increased (Fig. 5i; *P* = 0.033), while length of propagation was shortened (Fig. 5j; *P* = 0.044) and velocity was reduced (Fig. 5k; *P* = 0.012) by dexmedetomidine 15 mins after injection as against saline. However, CMMCs did not differ significantly in endotoxemic mice receiving 10% fat emulsion and those receiving propofol. This suppression of dexmedetomidine on CMMCs disappeared 24 h after administration.

### Dexmedetomidine, not propofol, depressed whole gastrointestinal motility in endotoxemic mice

**Fig. 6:**
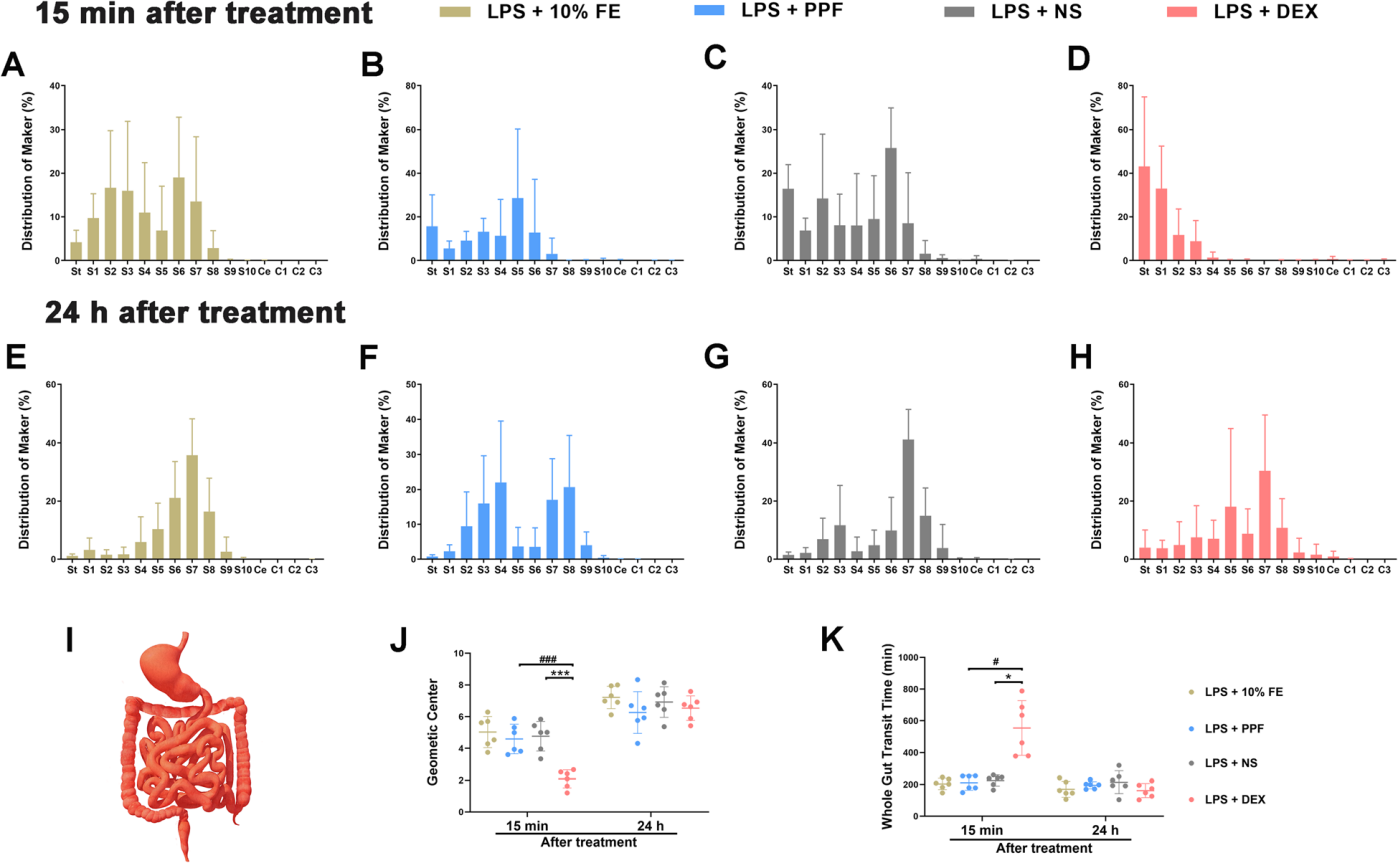
Effect of dexmedetomidine or propofol on the whole part of gastrointestinal motility in endotoxemic mice. n = 6 per group. Transit histogram for the distribution of non-absorbable fluorescein isothiocyanate through the intestinal segments 15 mins (**a**-**d**) and 24 h (**e**-**h**) after administration sedatives/vehicles (St, stomach; S, small intestine; Ce, cecum; C, colon). **i**. A schematic diagram of the whole gastrointestinal tract. 15 mins after treatment, dexmedetomidine depressed the whole gastrointestinal motility that showed smaller geometic center (**j**.) and longer latency of the first blue feces expulsion(**k**.). Results of these two tests were similar between groups 24 h after administration. Data were expressed as mean ± SD and analysed by one-way ANOVA or Kruskal–Wallis test. ^*^P < 0.05, ^***^P < 0.001, LPS + DEX vs LPS + NS. ^#^*P* < 0.05, ^###^*P* < 0.001, LPS + DEX vs LPS + PPF. LPS, lipopolysaccharide. FE, fat emulsion. PPF, propofol. NS, normal saline. DEX, dexmedetomidine

GIT and WGTT as sensitive methods to assess the motility of the whole GI tract (Fig. 6i). We checked the distribution of FITC-dextran in endotoxemic mice 15 mins (Fig. 6a-d) and 24 h (Fig. 6e-h) after administration of 10% fat emulsion, propofol, saline, and dexmedetomidine respectively. The dexmedetomidine group had smaller GC than the saline group (*P* = 0.0001) or propofol group (*P* = 0.0003) 15 mins after application (Fig. 6j). In respect of the WGTT, dexmedetomidine (554.5 ± 172.6 mins) significantly prolonged latency of the first blue feces expulsion 15 mins after administration compared with saline (224.7 ± 35.3 mins, *P* = 0.034) and propofol (210.3 ± 46.9 mins, *P* = 0.017) (Fig. 6k). However, propofol had suppression on neither GIT nor WGTT. What’s more, this inhibition of dexmedetomidine on the whole gastrointestinal motility had considerable abatement 24 h after injection.

## Discussion

Our results showed that deep sedation with dexmedetomidine, but not propofol suppressed motility of various parts of the GI tract including the stomach, small intestine, and colon in endotoxemic mice, whereas such inhibitory effects of dexmedetomidine recovered at 24 h after sedation. Additionally, dexmedetomidine led to heart rate reduction in endotoxemic mice.

Animal models of sepsis are generally divided into 3 categories: bacterial infection models, endotoxin models, and peritonitis models. Cecal ligation and puncture (CLP), a peritonitis model, has been considered a golden standard of sepsis research. However, this model requires an abdominal surgical procedure that may strikingly interfere with GI motility cause the induction of postoperative ileus [23]. Postoperative ileus is possible to obscure the effect of sedatives on GI motility in sepsis. Additionally, the LPS-induced endotoxin model was more stable compared to usage of bacteria, hence we injected 5 mg·kg^− 1^ LPS in mice to build a septic model.

Before comparing the effect of sedatives on GI motility in endotoxemic mice, the sedative depth of different doses of propofol and dexmedetomidine was assessed. Previous study demonstrated that the ED50 and ED95 of propofol for smooth insertion of the laryngeal mask airway were 2.9 mg·kg^− 1^ and 3.9 mg·kg^− 1^ respectively [24], which equaled to 35.67 and 47.97 mg·kg^− 1^ in mice based on human equivalent dose calculation scale [25]. And 50 mg·kg^− 1^ and 100 mg·kg^− 1^ propofol were used to evaluate the effect on GI motility [12]. Thus, we injected 40 mg·kg^− 1^ and 50 mg·kg^− 1^ propofol to examine the sedative effect in endotoxemic mice. In addition, 0.5 to 1 mg·kg^− 1^ dexmedetomidine had been reported for mice anesthesia [26]. ED50 of dexmedetomidine inhibited gastrointestinal transit was 40 μg·kg^− 1^ in rats, it may equal to 80 μg·kg^− 1^ for mice [25]. As a consequence, 40 μg·kg^− 1^ and 80 μg·kg^− 1^ dexmedetomidine was selected to assess sedative depth of mice in present study. Since 40 mg·kg^− 1^ propofol and 80 μg·kg^− 1^ dexmedetomidine induced comparable deep sedative in endotoxemic mice and the comfort and safety of patients who were undergoing mechanical ventilation ICU entailed deep sedation [27], we employed these doses in the present study. In line with the previous study, dexmedetomidine strongly decreased heart rate [28].

As for administration route, we used a single intraperitoneal injection in present study. Though intravenous injection is more in line with clinical practice, there are practical limitations associated with the technical difficulties of intravenous administration in mice due to their small size, especially in conscious mice [29]. It was not excluded that sedative drugs had directly implication on the gut, but we believed that even intravascular administration of drugs could also act on the gut as drugs would reach gut through blood circulation soon.

We tested GI motility 15 mins and 24 h after drug administration respectively. The maximum sedation depth was reached 15 min after drug administration, therefore we thought it was the right time to access the effect of sedatives on GI motor function. And the terminal half-life of single administration of 100 mg·kg^− 1^ propofol in the mouse blood is 140.8 ± 53.55 mins [30] and the elimination half-life of DEX is 2 ∼ 3 h. To figure out whether the inhibition of sedatives on GI motor function was sustained after metabolism of these sedatives, we conducted these GI motility tests 24 h after drug implement and found no sustained inhibition existed.

Although some human studies showed that GE [31] and GI motility [32] were uninfluenced by light or sub hypnotic propofol sedation, a human study in vitro founded a dose-dependent depression of propofol on gastric and colonic muscle [33]. Inada et al showed that 50 mg·kg^− 1^ propofol weakly repressed GE while 100 mg·kg^− 1^ propofol exhibited a marked inhibitory effect on GE and GIT in mice [12]. Thus, it indicated that propofol had a dose-dependent depression on GI motility. Our study explored that, in endotoxemic mice, 40 mg·kg^− 1^ propofol was enough to reach deep sedation while had little effect on GI motility. The mechanism of propofol effect on GI motility is complicated and is still a matter of debate. There are three types of GABA receptors (GABA_A_, GABA_B,_ GABA_C_) expressed in different regions of GI tract [34]. It was verified that GABA_A_ involved in the effect of propofol on GI motility [35]. Few studies explored the effect of propofol on GABA_B_ and GABA_C_ receptors in respect of GI motility. In a word, the mechanism of propofol on GI motility still needs to be investigated.

The antiperistatical effects in the current study that 80 μg·kg^− 1^ dexmedetomidine inhibited all segments of GI tract motor function of endotoxemic mice were consistent with studies in human [13] and animals [7]. Dexmedetomidine increased the frequency of CMMCs while decreased the propagation and velocity of CMMCs in our study. It was in line with the previous study that dexmedetomidine inhibited the guinea pig ileum peristalsis whereas increased the frequency of peristalsis waves in vitro. It might due to the incomplete peristalsis from mouth to anal that triggered an increased peristalsis frequency. Dexmedetomidine is a highly selective α2-adrenoceptor agonist, its inhibitory effect on ileum peristalsis could be prevented only by α2-adrenoceptor antagonist yohimbine instead of α1-adrenoceptor antagonist prazosin [7], which further indicated that the antiperistatical effect of dexmedetomidine may due to α2-adrenoceptor–mediated interruption of excitatory cholinergic pathways in the enteric nervous system [36] or activated inhibitory neural pathways [37]. The α2A subtype in the enteric nervous system was responsible for the suppression of medetomidine on GI motility [38, 39], it might have a potential role in the inhibition of dexmedetomidine on GI peristalsis. Besides, dexmedetomidine inhibited colon motility through a peripheral mechanism in present study, whether the central mechanism involved in this inhibition was speculative.

## Conclusion

In conclusion, at a comparable deep sedative level in endotoxemic mice, dexmedetomidine, but not propofol inhibited motilities of all parts of the GI tract, however, such inhibitory effects of dexmedetomidine disappeared after 24 h. So we could speculate that this side effect is short-term, while the prognosis of patients requiring long-term sedation with sedatives remains unknown. Additionally, dexmedetomidine produced obvious heart rate reduction in endotoxemic mice. These data indicated that both propofol and dexmedetomidine can be used in patients with sepsis, while dexmedetomidine should be used with caution in patients with heart disease or gastrointestinal motility disorder. However, take species differences into consideration, this finding needs more clinic investigation to be extrapolated to the situation in humans.

## Data Availability

The datasets used and/or analysed during the current study are available from the corresponding author on reasonable request.

## Abbreviations

LPS: Lipopolysaccharide
ICU: Intensive care unit
GI: Gastrointestinal
ARRIVE: Animal research: reporting of in vivo experiments
GE: Gastric emptying
SIT: Small intestinal transit
CMMCs: Colonic migrating motor complexes
GIT: Gastrointestinal transit
WGTT: Whole gut transit time
SD: Standard deviation
CLP: Cecal ligation and puncture
